# Quality of Life and Financial Burden in Duchenne Muscular Dystrophy in Greece: Insights into Health System Performance in the Post-Pandemic Context

**DOI:** 10.3390/healthcare13222835

**Published:** 2025-11-08

**Authors:** Eleni Katsomiti, Catherine Kastanioti, Elisabeth Chroni, George Mavridoglou, Evangelos Pavlou

**Affiliations:** 1 Department of Business Administration and Organizations, University of Peloponnese, 24100 Kalamata, Greece; a.kastanioti@uop.gr; 2 Neurology Department, Medical School, University of Patras, 26504 Patra, Greece; 3 Department of Accounting and Finance, University of Peloponnese, 24100 Kalamata, Greece; ge.mavridoglou@go.uop.gr; 4 2nd Department of Pediatrics, University General Hospital AHEPA, 54636 Thessaloniki, Greece; espavlou@auth.gr

**Keywords:** Duchenne Muscular Dystrophy, health-related quality of life, COVID-19, rare diseases, pediatric health policy, PedsQL, healthcare delivery, health policy

## Abstract

**Background:** The COVID-19 pandemic disrupted healthcare systems, disproportionately affecting individuals with rare diseases. This study explores the health-related quality of life and financial burden in the post-pandemic context among children with Duchenne Muscular Dystrophy and their families in Greece, providing insights into health system performance. **Methods:** A multicenter, cross-sectional study was conducted in two neuromuscular clinics in Greece. Fifty families (response rate 67%) completed standardized quality of life instruments (PedsQL™ 4.0 Generic Core Scale; PedsQL™ 3.0 DMD Module) and a socioeconomic questionnaire. Descriptive and correlation analyses assessed associations between functional status, financial strain, and psychosocial indicators. **Results**: Children with Duchenne Muscular Dystrophy reported moderate-to-severe reductions in physical and emotional well-being, and substantial out-of-pocket expenditures. Families with greater financial strain or wheelchair-dependent children had significantly lower health-related quality of life scores. Insurance coverage was positively associated with emotional and psychosocial functioning. **Conclusions**: Greek families living with Duchenne Muscular Dystrophy continue to face significant financial and psychosocial challenges in the post-pandemic period. While the cross-sectional design does not allow causal attribute to COVID-19 pandemic, the results underscore the need to strengthen financial protection, coordinated multidisciplinary care, and equitable access to support services for rare disease management.

## 1. Introduction

Duchenne Muscular Dystrophy (DMD) is a rare, severe, and progressive X-linked neuromuscular disorder that primarily affects boys, leading to muscle degeneration, loss of ambulation, and premature death due to cardiac or respiratory complications [1,2,3]. With an estimated global prevalence of 19.8 per 100,000 live male births, DMD is the most common and debilitating dystrophinopathy [4]. Despite advances in multidisciplinary care—including corticosteroids, physiotherapy, and novel molecular therapies [5,6,7,8,9,10,11,12,13] life expectancy has only modestly improved [14,15,16], while families continue to face profound challenges beyond the clinical course of the disease.

Assessing the health-related quality of life (HRQoL) and financial burden associated with DMD has become increasingly important in rare disease research and policy. While global Organizations such as those of the World Health Organization [17] and the European Commission [18] advocate for patient-centered, equity-oriented approaches, studies on DMD often emphasize clinical or cost-related aspects without adequately linking these to the lived experiences of patients and caregivers. Furthermore, international cost-of-illness studies [19,20,21,22,23,24,25,26,27,28,29] conducted in countries such as Spain, Portugal, Italy, Denmark and Japan have demonstrated substantial economic strain but were largely undertaken before the COVID-19 pandemic and rarely integrated validated, disease-specific HRQoL instruments [30,31,32].

In Greece, research on DMD remains scarce. The country’s health system, characterized by fragmented reimbursement mechanisms and limited financial protection for families affected by rare diseases, faces additional pressures in the post-pandemic era. The pandemic has exacerbated pre-existing gaps in service continuity, rehabilitation access, and social support, highlighting the urgent need to examine how clinical severity, caregiving demands, and financial strain interact to influence family well-being.

Our study addresses these gaps by providing the assessment of HRQoL using validated Greek versions of the PedsQL™ 4.0 Generic Core and PedsQL™ 3.0 DMD Module instruments [33,34] and socioeconomic burden among children with DMD in Greece’s post-pandemic health system. By focusing on the intersection of socioeconomic, clinical, and psychosocial dimensions, this study provides evidence to inform equitable rare disease management and health policy planning in Greece and comparable healthcare settings.

## 2. Materials and Methods

### 2.1. Study Design and Patient Population

This multicenter, cross-sectional study was conducted between September 2022 and July 2023 among pediatric patients with DMD and their families in Greece.

At the design stage, an informal nationwide patient registry of the patients’ organization MDA-Hellas was consulted to identify eligible participants, yielding 125 boys aged 5–18 years with a genetically confirmed diagnosis of DMD. Within Greece’s four specialized neuromuscular centers, two—AHEPA University Hospital of Thessaloniki and the University General Hospital of Patras—consented to participate, as they manage the largest patient populations. In total, 75 patients were invited to join the study, representing 60% of all registered cases. Families were approached during routine medical visits to minimize travel burden. Of the 75 families contacted, 50 agreed to participate (response rate: 67%). The main reasons for non-participation were lack of interest, unwillingness to travel, and outdated contact information. The final sample represented the majority of registry-listed pediatric DMD patients in Greece, ensuring reasonable national representativeness. No formal sample size calculation was performed, as the study was exploratory and primarily descriptive in nature.

Data were collected using structured questionnaires administered either in person at the study sites or remotely via telephone or videoconference, as appropriate. Two instruments were developed for this study:

(a) Parental questionnaire: included demographic, clinical, socioeconomic, and caregiver-related variables, along with the validated PedsQL™ 4.0 Generic Core Scales and PedsQL™ 3.0 DMD Module (proxy version).
(b) Child questionnaire: included the PedsQL™ self-report scales, completed by children aged 8–18 years where feasible. Children aged 5–7 years did not complete self-reports.

Trained researchers assisted families, when necessary, by reading items aloud and recording responses. In cases where questionnaire items were missing, the following protocol was applied: first, the instrument manual [35] was consulted; second, the missing items were managed according to the prescribed guidelines. When more than 50% of items on a given scale were completed, the mean of the completed items was imputed; otherwise, the scale score was treated as missing.

The study was approved by the Institutional Review Boards of the University of Peloponnese (prot.no. 11415/31-5-2023), AHEPA Hospital (prot.no. 239/16-5-2022), and Patras University Hospital (prot.no. 246/31-5-2022). Written informed consent was obtained from parents or legal guardians of all participants, and assent was obtained from children ≥ 8 years where appropriate.

### 2.2. Measures

HRQoL was assessed using two validated instruments: the Pediatric Quality of Life Inventory™ (PedsQL™) 4.0 Generic Core Scales-GR and the disease-specific PedsQL™ 3.0 DMD Module-GR. The validated Greek versions of the PedsQL™ 4.0 Generic Core Scales [33] and PedsQL™ 3.0 DMD Module [34] were used, both of which have demonstrated strong psychometric properties in previous studies. In the current sample, internal consistency reliability was also assessed. Cronbach’s α coefficients exceeded 0.70 across all subscales of the PedsQL™ 4.0 GC and 0.80 for the PedsQL™ 3.0 DMD Module, confirming satisfactory internal consistency.

The PedsQL™ 4.0 GC is designed to differentiate between children with chronic health conditions and their healthy peers, offering a general assessment of HRQoL across physical, emotional, social, and school functioning domains. In contrast, the PedsQL™ 3.0 DMD Module allows for more disease-specific evaluation, facilitating comparisons in HRQoL among subgroups of DMD patients based on age, disease stage, and even cultural or ethnic background. The PedsQL™ 4.0 Generic Core Scales comprise 23 items across four domains: Physical Functioning (8 items), Emotional Functioning (5 items), Social Functioning (5 items), and School Functioning (5 items). A Psychosocial Health Summary Score is computed as the mean of the Emotional, Social, and School subscales. The instrument includes age-appropriate self-report (ages 5–18) and proxy-report formats (ages 2–18), with higher scores indicating better HRQoL. The PedsQL™ 3.0 DMD Module assesses disease-specific HRQoL and includes 18 items grouped into four domains: Daily Activities (5 items), Treatment Barriers (4 items), Worry (6 items), and Communication (3 items). It is available in child self-report formats for ages 8–12 and 13–18 years and proxy versions for ages 5–7, 8–12, and 13–18 years. All items across both instruments are scored using a 5-point Likert scale (0 = never a problem to 4 = almost always a problem), reverse-scored, and transformed to a 0–100 scale, with higher scores reflecting better perceived quality of life. The instruments were administered based on the child’s age and cognitive capacity. Together, they offer a comprehensive assessment of general and condition-specific quality of life (HRQoL).

The socioeconomic questionnaire was adapted based on European and international health economics frameworks for out-of-pocket health expenditures [36,37,38,39,40]. The socioeconomic questionnaire included questions about household income, receipt of a state allowance, unexpected or on a regular basis out-of-pocket costs concerning medical or non-medical expenses related to the child’s condition, impact on parents’ employment, public or private insurance and benefits covered by insurance. Internal consistency was not computed given the multidimensional (non-scale) nature of the questionnaire.

Household income (€) was grouped in three categories with lower and upper limits. Regular out-of-pocket costs (€) were calculated as monthly reports × 12, unexpected costs were recorded as lump sums in the past 12 months. Financial burden was expressed both in absolute values (€) and relative to household income (%). Employment impact was coded as full-time, part-time, unemployed due to caregiving, or unemployed unrelated to caregiving. The status of insurance was indicated as public, private or no insurance and regarding the benefits covered by insurance the participants indicated whether they received it or not. As for the place of residence, there were three options: “Attica”, “Thessaloniki” and “other”. The socioeconomic questionnaire captured, also, information on household income (€) and state allowance receipt (€) (extra-institutional care allowance €846/month, social solidarity allowance €338/month or no allowance).

### 2.3. Statistical Analysis

Data was analyzed using IBM SPSS 29.0.1. Descriptive statistics and correlation analyses were applied to examine associations on patient demographics, clinical features, and socioeconomic characteristics presented as means, medians, standard deviations, ranges, and percentages where appropriate. Bivariate analyses (Mann–Whitney U, Kruskal–Wallis, and Spearman’s correlations) explored associations between HRQoL or financial burden and explanatory variables, respectively. The Mann–Whitney U test was used for comparisons between two independent groups, while the Kruskal–Wallis test was applied for comparisons across three or more groups. The significance level of *p* < 0.05 was considered statistically significant for all analyses. Agreement between child self-reports and parent proxy-reports was assessed using intraclass correlation coefficients (ICCs). The management of missing values with regard to the PedsQL measures was based on the pertinent scoring instructions [35]. With regard to the socioeconomic questionnaire, no missing values were encountered, as this research was of an interview-type nature.

Due to the limited sample size (n = 50), multivariable regression models were not conducted in order to avoid overfitting. Accordingly, it is imperative to interpret the findings as exploratory and hypothesis-generating, as opposed to a confirmatory approach.

## 3. Results

### 3.1. Socio-Demographic and Clinical Characteristics

Fifty children with DMD with a mean age of 12.5 years old (SD = 3.0) and a mean age at diagnosis of 3.6 years old (SD = 2.8), were included. Nearly one-third (30%) were non-ambulatory, 46% were receiving corticosteroid therapy, and 54% were undergoing physiotherapy. Two-thirds (68%) had a positive family history of DMD. A detailed breakdown of demographic and clinical features is provided in Table 1.

### 3.2. Financial Burden and Socioeconomic Profile

Financial data were available for 43 families. Most participants lived in urban regions (Attica and Thessaloniki), with the remainder distributed across other Greek prefectures. Approximately three-quarters (74%) reported an annual household income below €25,000. More than one in four families (28%) faced monthly out-of-pocket costs equal to or exceeding the value of their state allowance. Unexpected annual health-related costs were common (particularly in families with wheelchair-dependent children), in some cases representing up to 25% of household income.

**Figure 1:**
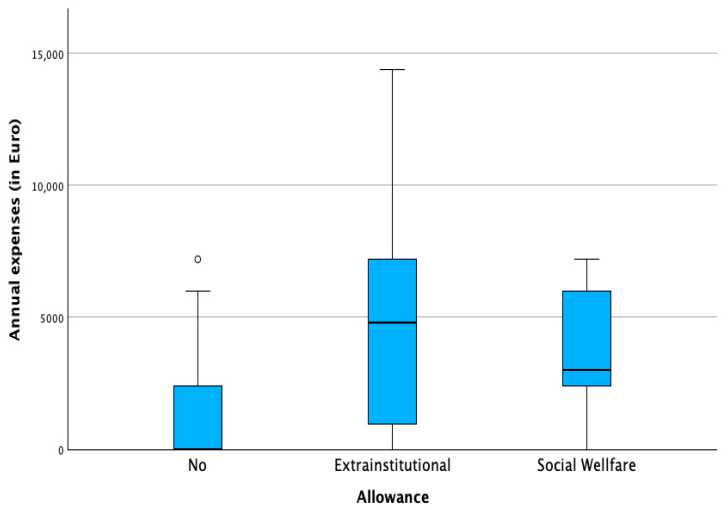
Annual expenses in regard to state allowance. (◦ potential outlier).

**Figure 2:**
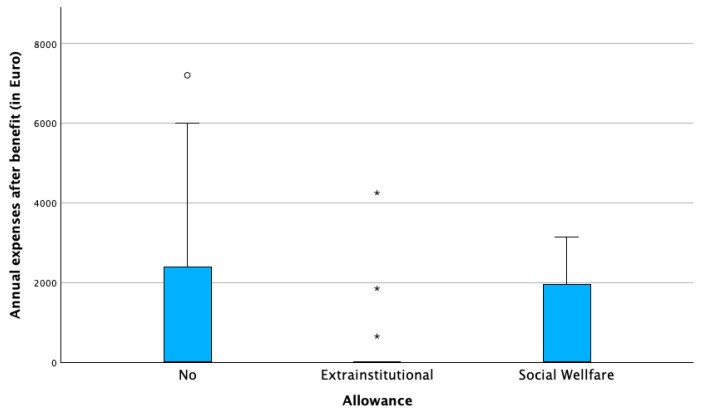
Annual expenses after benefit of state allowance in euros (€). (◦ potential outlier, * extreme outlier).

Allowance status played a key role. Families receiving the extra-institutional allowance (€846/month) reported the highest absolute expenses and the most frequent unexpected costs. While this allowance reduced net financial burden, in 18% of families, expenses still exceeded the total allowance. Families receiving the lower social allowance (€338/month) consistently reported uncovered monthly costs. Those without allowances incurred fewer direct expenses but remained financially vulnerable. Table 2 shows the number of patients towards the receipt of the allowance and Figure 1 and Figure 2 illustrate the distribution of annual expenses towards the distribution of the state allowance, before and after the benefit of the allowance.

To further explore the determinants of financial burden among participating families, factors associated with expenditures related to the child’s condition were examined (Table 3). The results indicated the following: (a) Unexpected annual expenses (€). These were significantly associated with three factors: child’s age, wheelchair dependency, receipt of a state allowance, and when the unexpected costs were expressed as percentage of the income, the factor the geographic region of the family’s residence was added. (b) Regular annual expenses (€). Calculated by multiplying reported monthly out-of-pocket costs by 12, these expenses were also associated with allowance status and regional residence. Interestingly, a different pattern emerged compared to unexpected costs.

Further analysis revealed regional differences between urban and rural respondents. Families living in Athens or Thessaloniki reported higher out-of-pocket expenses, likely due to more frequent access to specialized care and therapies. In contrast, families in rural areas reported lower direct costs.

Similarly, high-cost, one-time expenditures such as home or vehicle modifications to accommodate wheelchair access were observed more frequently in urban areas compared to rural ones.

### 3.3. Health-Related Quality of Life Outcomes

Using the validated Greek versions of PedsQL™ 4.0 GC and PedsQL™ 3.0 DMD Module, children reported moderate to low HRQoL scores. Total scores on the Generic Core Scales (GC) averaged approximately 60 for child self-reports and 58 for parent-proxy reports, indicating substantial impairments in physical and psychosocial functioning compared to normative values (>80). In contrast, mean scores on the DMD Module were somewhat higher (≈70), reflecting relative adaptation to disease-specific challenges. Child and parent reports showed high concordance, with intraclass correlation coefficients of 0.91 for the GC and 0.87 for the DMD Module. Detailed results are presented in Table 4 and Table 5.

### 3.4. Associations Between Socioeconomic and Clinical Variables and HRQoL Domains

The findings presented in Table 6 are based on non-parametric statistical tests (Kruskal–Wallis and Mann–Whitney U, α = 0.05), which revealed significant differences in HRQoL scores across various socioeconomic and clinical categories.

One of the most prominent findings relates to the receipt of state allowances. Families receiving state allowances, particularly the extra-institutional allowance reported significantly lower HRQoL reflecting greater disease severity and financial stress. Furthermore, regular or unexpected annual out-of-pocket expenditures were inversely correlated with total HRQoL scores, particularly in the emotional, treatment, and daily activities domains. Regarding functional status, wheelchair dependency was consistently linked to lower HRQoL, especially in the physical and worry domains. In contrast, access to public or private health insurance was associated with higher emotional, social, and psychosocial functioning scores. Parental employment status and educational level were not significantly associated with HRQoL outcomes. Child age significantly influenced HRQoL, especially in the physical health domain (PedsQL™ 4.0 GC) and worry domain (PedsQL™ 3.0 DMD Module). Together, these findings demonstrate that both clinical progression and inadequate financial protection are major determinants of reduced quality of life among families affected by DMD in Greece.

## 4. Discussion

This study provides the first integrated assessment of the socioeconomic and psychosocial impact of DMD in Greece during the post-pandemic period. Our findings highlight a dual burden on affected families, characterized by substantial financial strain and reduced HRQoL. Using the validated Greek versions of the PedsQL™ 4.0 Generic Core [33] and PedsQL™ 3.0 DMD Module [34], children consistently reported moderate to low HRQoL scores, which were lower than those observed in healthy peers [34,41] and in children with other chronic conditions [34,41]. Overall, these findings confirm that Greek children with DMD experience significantly lower HRQoL than both healthy peers and children with other chronic conditions, emphasizing the profound physical and psychosocial burden of the disease on affected families.

Most households reported low-to-middle incomes, high caregiving demands, and limited insurance coverage. Although state allowances offered partial relief, they were insufficient to offset recurring and unexpected out-of-pocket expenditures, particularly among non-ambulatory children and single-income families. These socioeconomic challenges are likely to contribute to the reduced HRQoL observed among children with DMD and underscore the multifaceted burden experienced by families.

Moreover, our results demonstrate that economic hardship is strongly intertwined with psychosocial well-being. Higher out-of-pocket expenditures, limited financial protection, and dependence on allowances were all associated with lower emotional and overall HRQoL scores. Families experiencing severe disease progression or greater caregiving intensity reported the lowest HRQoL, indicating that clinical factors alone do not account for the difficulties faced. Instead, socioeconomic vulnerability and inadequate support mechanisms play a decisive role in shaping family well-being in DMD. This pattern is consistent with the descriptive trends observed in our data and further underscores the strong link between financial strain and HRQoL. Overall, the results emphasize the need to approach rare disease management not only from a clinical perspective but also as a socioeconomic and mental health issue.

When compared with other European studies, the Greek data reveal both common and context-specific patterns. As in Southern Europe [25,26,27], families experience high expenditures, often absorbing a significant share of care expenditures privately. However, Greece differs in its limited reimbursement structure and reliance on informal caregiving, resulting in greater dependence on family support networks.

Within-country variations further highlight regional inequities in service access. Families living in urban areas (Athens and Thessaloniki) reported higher total healthcare expenditures, reflecting greater access to specialized services and equipment. Conversely, rural families reported lower direct costs but more limited access to multidisciplinary care, likely due to service availability and travel barriers.

Placing these findings in an international context provides additional insight into both shared and unique aspects of the Greek experience. HRQoL scores in our cohort were comparable to those reported in Switzerland and Australia [42,43], slightly lower than in China [44], and somewhat higher than in Canada and the USA [45,46,47]. Psychosocial functioning appeared relatively favorable, likely reflecting strong family cohesion and adaptive coping typical of Mediterranean cultures. This resilience, despite structural weaknesses, underscores the cultural importance of family-based care but also reveals the systemic gaps left unaddressed by formal health and welfare services.

By linking validated HRQoL outcomes with socioeconomic indicators, our study extends existing evidence from European cost-of-illness analyses, which rarely integrate psychosocial dimensions. The findings identify three priorities for policy and service reform, strengthening financial protection through better-targeted allowances and comprehensive insurance coverage for non-reimbursed therapies, equipment, and home modifications, expanding access to multidisciplinary care—including physiotherapy, psychological counseling, and social support—especially in rural areas and supporting family caregivers, particularly mothers, through flexible employment policies and respite care programs to prevent financial and emotional exhaustion. Such measures would align Greek rare disease policy with broader European initiatives aimed at reducing inequities and improving quality of life for families affected by chronic and rare conditions.

Several limitations should be acknowledged. The cross-sectional design precludes causal inference, and the relatively small sample (n = 50) and limited regional coverage constrain generalizability. Additionally, the absence of a pre-pandemic comparison group prevents direct evaluation of COVID-19’s impact. Nonetheless, the study’s rigorous methodology and use of validated instruments provide a robust descriptive picture of post-pandemic challenges in DMD care.

Overall, this research advances understanding of how financial stress, caregiving demands, and clinical severity intersect to shape family well-being in DMD. It situates the Greek experience within a broader Southern European welfare context characterized by fragmented social protection. The study thus contributes actionable evidence for strengthening resilience, financial equity, and psychosocial support in rare disease care—both in Greece and in comparable health systems.

## 5. Conclusions

This study provides context-specific evidence on the socioeconomic and psychosocial burden experienced by families of children with DMD in Greece during the post-pandemic period. Families with greater out-of-pocket expenditures, limited insurance coverage, or reliance on state allowances reported significantly lower HRQoL, highlighting the direct link between financial vulnerability and psychosocial well-being. The findings emphasize that the challenges faced by Greek families affected by DMD extend beyond clinical management. Fragmented reimbursement, regional inequalities in service access, and the predominance of unpaid caregiving—often by mothers—contribute substantially to household stress and reduced quality of life. These results mirror patterns seen across Southern Europe, reinforcing the need for stronger financial protection mechanisms and family-centered support policies in resource-constrained welfare systems. The study’s cross-sectional design and modest sample size limit causal inference and generalizability. Moreover, the absence of a pre-pandemic comparator precludes direct attribution of findings to COVID-19. Nonetheless, the use of validated instruments and detailed socioeconomic data strengthens the reliability and contextual relevance of the results. Overall, strengthening financial assistance, expanding access to multidisciplinary rehabilitation and psychosocial care, and supporting family caregivers are key priorities for building more equitable and resilient rare disease policies. The Greek experience contributes valuable insights for other countries facing similar constraints, offering a framework for integrating economic and HRQoL outcomes into rare disease policy and practice.

## Figures and Tables

**Table 1:** Demographic and clinical characteristics.

Variable	Mean	SD	Range
Age at onset (years)	3.58	2.83	0–10
Age at time of the evaluation (years)	12.52	2.97	8–19
**Variable**	**N**	**%**	
Age distribution(years)			
5–7	9	18.0%	
8–12	24	48.0%	
13–18	17	34.0%	
Non-ambulatory patients	15	30.0%	
Current steroid use	23	46.0%	
Physiotherapy	27	54.0%	
Family history	34	68.0%	
**Place of residence**			
Attica	11	22%	
Thessaloniki	16	32%	
Other	23	46%	

**Table 2:** Socioeconomic characteristics (Distribution of patients according to the receipt of allowance).

		Extra Institutional Allowance	Social Wellfare Allowance	No Allowance
		22	9	12
Monthly out-of-pocket expenditures	yes	17	9	5
	no	5	0	7
Monthly out-of-pocket-money not covered by amount of the allowance		3	4	5
Use of wheelchair	no	6	8	12
	partly	4	0	0
	always	12	1	0
Physiotherapy covered by public or private insurance	yes	15	5	6
	no	7	4	6
Annual income	Up to €15.000	7	3	3
	€15.001–€25.000	9	4	6
	More than €25.001	6	2	3
Place of residense	Attica	8	0	0
	Thessaloniki	6	3	6
	Other	8	6	6
Mother’s labour	full-time	10	5	6
	part-time	2	2	1
	no	8	2	5
	pension	2	0	0

**Table 3:** Bivariate analysis between regular or unexpected annual costs and the affecting factors.

Annual Unexpected Costs	Annual Unexpected Costs as Percentage of Annual Income	Annual Regular Expenses (Monthly out-of-Pocket Costs × 12)
Age	Age	
Wheelchair	Wheelchair	
Allowance	Allowance	Allowance
	Region	Region

**Table 4:** PedsQL™ 4.0 Generic Core Scale scores: child self-reports vs. parent proxy reports.

	5–7 (Years)		8–12 (Years)		13–18 (Years)	
	Mean	SD	Mean	SD	Mean	SD
**Child self–report**			(Ν = 14)		(Ν = 11)	
Total Score GC			59.86	15.10	61.65	18.47
Physical Health Summary Score			46.21	25.04	39.77	29.42
Emotional Score			67.14	15.65	72.39	17.68
Social Score			66.79	14.22	71.82	17.65
School Score			67.50	19.19	76.70	17.59
Psychosocial Score			67.14	12.06	73.64	14.66
**Parent proxy report**	(Ν = 7)		(Ν = 20)		(Ν = 15)	
Total Score GC	65.83	20.94	60.82	16.90	49.47	20.42
Physical Health Summary Score		30.77	48.44	26.41	25.22	29.15
Emotional Score	75.00	21.41	69.25	18.23	64.64	27.42
Social Score	64.11	20.70	62.00	23.92	56.61	17.64
School Score	71.43	19.30	71.00	17.74	75.23	19.12
Psychosocial Score	70.22	19.01	67.42	15.75	64.83	19.53

**Table 5:** PedsQL™ 3.0 DMD module scores: child self-reports vs. parent proxy reports.

	5–7 (Years)		8–12 (Years)		13–18 (Years)		All	
	Mean	SD	Mean	SD	Mean	SD	Mean	SD
**Child self–report**			(Ν = 18)		(Ν = 13)		(Ν = 31)	
Total (18)			70.85	11.55	71.02	22.92	70.92	16.91
Daily Activities (5)			74.44	16.71	67.69	29.41	71.61	22.71
Treatment Barriers (4)			75.46	21.63	71.35	22.69	73.82	21.77
Worry (6)			66.69	17.56	77.63	15.17	71.28	17.23
Communication (3)			66.67	21.77	63.46	38.12	65.32	29.19
**Parent proxy report**	(Ν = 8)		(Ν = 24)		(Ν = 16)		(Ν = 48)	
Total (18)	76.18	20.69	66.21	18.73	56.74	25.62	64.71	22.14
Daily Activities (5)	56.88	33.16	60.83	25.62	50.00	38.21	56.56	31.22
Treatment Barriers (4)	82.81	21.84	71.20	20.62	63.28	32.02	70.48	25.59
Worry (6)	84.38	19.13	64.90	20.79	56.04	28.49	65.19	24.82
Communication (3)	84.38	16.33	69.10	30.74	59.90	36.80	68.58	31.66

**Table 6:** Bivariate analysis results-variables associated with HRQoL domains in children with DMD.

x/y	Allowance *	Annual Unexpected Expenses **	Provision by Insurance **	Monthly Expenses **	Use of Wheelchair **	Age Group *	Corticoids **
Daily activities	Yes	Yes			Yes		
Treatment	Yes			Yes			
Worry	Yes	Yes			Yes	Yes	Yes
Communication	Yes						
Total Score DMD	Yes						
Physical Health Summary Score	Yes	Yes			Yes	Yes	
Emotional Score	Yes		Yes	Yes			
Social Score			Yes				
School Score	Yes						
Psychosocial Score	Yes		Yes				
Total Score GC	Yes	Yes	Yes		Yes		

* Kruskal–Wallis test was applied for categorical variables with >2 groups (e.g., age groups, allowance status, region). ** Mann–Whitney U test was applied for dichotomous variables (e.g., wheelchair use, corticosteroid treatment, insurance status).

## Data Availability

The dataset used and analyzed in this study is available from the corresponding author. The data are not publicly available due to ethical restrictions.
